# Testing the Effectiveness of a Mobile Smartphone App Designed to Improve the Mental Health of Junior Physicians: Protocol for a Randomized Controlled Trial

**DOI:** 10.2196/58288

**Published:** 2024-09-19

**Authors:** Lauren Lai, Samineh Sanatkar, Andrew Mackinnon, Mark Deady, Katherine Petrie, Rosie Lipscomb, Isabelle Counson, Rohan Francis-Taylor, Kimberlie Dean, Samuel Harvey

**Affiliations:** 1 Black Dog Institute Randwick Australia; 2 Discipline of Psychiatry and Mental Health School of Clinical Medicine University of New South Wales, Sydney Kensington Australia

**Keywords:** junior physicians, digital mental health, smartphone app, depression, mobile phone

## Abstract

**Background:**

Shift (Black Dog Institute) is the first mobile health smartphone app created to support the mental health of junior physicians. Junior physicians experience demanding work conditions, leading to high levels of psychological distress and burnout. However, they are often concerned about the potential career impacts of seeking mental health support. The confidentiality and ease of access of digital interventions may be particularly suited to address these concerns. The Shift app provides therapeutic and psychoeducational content and strategies contextualized for the specific needs of physicians in training. App content includes information on mental health, help seeking, mindfulness, and common workplace-related concerns of junior physicians.

**Objective:**

This study aims to test, at scale, the effectiveness of Shift among junior physicians working in Australia using a randomized controlled trial design. The primary aim is to examine whether junior physicians using Shift experience a reduction in depressive symptoms compared with a waitlist control group. The secondary aim is to examine whether the app intervention group experiences improvements in anxiety, work and social functioning, help seeking, quality of life, and burnout compared with the control group.

**Methods:**

A total of 778 junior physicians were recruited over the internet through government and nongovernment medical organizations across Australia, as well as through paid social media advertisements. They were randomly allocated to one of 2 groups: (1) the intervention group, who were asked to use the Shift app for a period of 30 days, or (2) the waitlist control group, who were placed on a waitlist and were asked to use the app after 3 months. Participants completed psychometric measures for self-assessing mental health and wellbeing outcomes, with assessments occurring at baseline, 1 month after completing the baseline period, and 3 months after completing the baseline period. Participants in the waitlist control group were asked to complete an additional web-based questionnaire 1 month after receiving access to the app or 4 months after completing the baseline survey. Participants took part in the study on the internet; the study was completely automated.

**Results:**

The study was funded from November 2022 to December 2024 by the New South Wales Ministry of Health. Data collection for the study occurred between January and August 2024, with 780 participants enrolling in the study during this time. Data analysis is underway; the effectiveness of the intervention will be estimated on an intention-to-treat basis using a mixed-model, repeated measures analysis. Results are expected to be submitted for publication in 2025.

**Conclusions:**

To the best of our knowledge, this is the first randomized controlled trial to examine the effectiveness of a mobile health smartphone app specifically designed to support the mental health of junior physicians.

**Trial Registration:**

Australia and New Zealand Clinical Trials Registry ACTRN12623000664640; https://tinyurl.com/7xt24dhk

**International Registered Report Identifier (IRRID):**

DERR1-10.2196/58288

## Introduction

### Background

The mental health and wellbeing of junior physicians is of increasing concern, with the term *junior physician* applying to physicians who have worked for 5 to 11 years or more after they enter the workforce [1]. As they navigate the transition from medical school to the workforce, junior physicians experience high work demands. Junior physicians must balance highly demanding working conditions and expectations of high performance with the completion of examinations, long working hours, often unsupportive work environments, and relative inexperience in clinical practice [1].

These stressors can place junior physicians at a heightened risk of mental and physical ill-health, which can be particularly impactful at a time that is critical for establishing future health, life goals, and career progression [2-4]. A comprehensive 2013 study of physicians in Australia found that junior physicians showed higher levels of psychological distress, burnout, and thoughts of suicide compared with more experienced physicians [5]. Psychological distress in junior physicians remains high in the post–COVID-19 era; a 2022 study of UK-based junior physicians reported poor mental health in those sampled (429/456, 94.1% total participants), with 45.2% (194/429) of the participants scoring highly on depression symptoms and 63.2% (271/429) scoring highly on anxiety symptoms [6]. The high prevalence of mental health problems is compounded by the physicians’ low rates of seeking help for suicidal thoughts and mental health concerns [7]. A range of barriers to seeking help exist for physicians, including self-stigmatization, concerns about being reported to medical regulators, and subsequent negative career repercussions [8].

Mobile health smartphone apps (mHealth apps) have the potential to provide free, 24×7, confidential support by providing educational content and skills training. They can aid in the prevention and early intervention of mental illness by providing easily accessible evidence-based information, such as exercises teaching the basics of cognitive behavioral therapy and acceptance and commitment therapy. Furthermore, they can provide avenues, including clinical services, for users to seek mental health support for those with more severe psychological distress. Presenting this information in the form of an app may circumvent the common barriers to seeking help and avoid the impact of stigma. This is because mental health information and support can be provided in a confidential manner that is unrelated to users’ places of work. Previous studies have found mHealth apps to increase resilience and decrease symptoms of depression in other high-risk workplace populations such as ex-soldiers and male-dominated industries including agriculture and mining [9,10]. While there have been a few efforts to develop mHealth apps for health care workers more generally [11,12], specific digital support options for the junior physician population remain lacking.

The Shift app was funded by and developed as part of a 10-step plan by the local Australian state government, the New South Wales (NSW) Ministry of Health, to improve the wellbeing and support available for junior physicians. As part of the scoping assessment for the app, the research team conducted interviews with 12 junior physicians to examine the acceptability of an mHealth app for mental health in this population. The majority of participants surveyed were “very or somewhat interested” in an mHealth app specifically designed to support the mental health and wellbeing of physicians who are early in their careers [13]. They agreed that the main barrier to app use was a lack of time and concerns about confidentiality [13].

Therefore, the Shift app was purposely created to target the unique circumstances of junior physicians. The app specifically incorporates modules and content aimed at the relevant stressors of this group such as shift work, long working hours, dealing with consultations, and where to seek help as a medical professional. Each module includes a scenario of a junior physician facing an emotional or a professional problem, with solutions geared toward these physicians in training. It is hoped that covering a wide range of common problems faced by junior physicians will help to meet the varying needs of individual users, including tools to stay mentally healthy, greater education and awareness of mental health management, and encouraging the users to actively seek mental health help.

To ensure that the preferences and needs of junior physicians were met, the Shift app was developed in multiple phases, with a user-centric design process. An overview of the multiphase design process can be seen in Textbox 1. An in-depth report of phases 1 to 4 can be found in Counson et al [13]; phase 5 is detailed in Sanatkar et al [14]. Notably, a 2020 uncontrolled evaluation of the Shift app with 222 junior physicians across NSW found significant reductions in symptoms of depression and anxiety after 1 month of app use [14]. While preliminary findings are promising, a nationwide randomized controlled trial (RCT) with junior physician participants is required to reliably establish the app’s effectiveness.

**A summary of the different phases that were completed in the process of designing the Shift app.**

**Phase 1: qualitative interviews**

- Needs assessment through qualitative interviews with 12 junior physicians in New South Wales (NSW). Topics surveyed included attitudes toward an app, desired app contents, and barriers to app use.

**Phase 2: prototype development**

- A prototype of the app was developed, with content written on the basis of recommendations from Phase 1 and in line with evidence from recent literature.

**Phase 3: pilot-testing**

- The app prototype was pilot-tested with 22 junior physicians who were recruited from one rural and one metropolitan hospital in NSW. Results revealed issues with the organization of the app content, but overall usability ratings were high.

**Phase 4: app redevelopment**

- The app was redeveloped to increase user engagement, with changes made to improve layout and design, increase personalization, update the clinical contents, and adopt more meaningful and relatable wording. This phase was informed by three user consultation workshops with 51 junior physicians in NSW.

**Phase 5: uncontrolled evaluation study**

- An uncontrolled evaluation study of the Shift was conducted in 222 junior physicians working in NSW during the COVID-19 pandemic. Depressive and anxiety symptoms significantly decreased after one month of app use.

**Phase 6: naturalistic evaluation**

- The Shift app was made freely available to the public, with a reduced research component where participants were able to opt-out of data collection. This enabled further interpretation of the effectiveness of the app in a naturalistic setting and to inform future improvements and Shift’s ongoing safety and quality.

**Phase 7: minor redesign**

- A minor redesign of the app was performed in preparation for a nationwide rollout of the app in a randomized controlled trial, including updating the content to be relevant to a nationwide audience, and design changes to increase usability. For further detail, see the Intervention section of this paper.

### Outcomes

This study is a national RCT with junior physicians to test the effectiveness of the Shift app. Specifically, we will investigate whether junior physicians who are allocated to the intervention group experience a significantly greater reduction in the symptoms of depression than those who were allocated to the control group (Primary and Secondary Outcomes: Depression section) after a period of 30 days. We will also compare intergroup differences in anxiety symptoms, work and social functioning, quality of life, burnout, and help-seeking intentions and behaviors (Primary and Secondary Outcomes section). Finally, we will assess the suitability of the app and its contents using feedback from junior physicians.

## Methods

### Study Design

The study is a 2-arm parallel RCT. Randomization is not stratified and will automatically occur when a participant completes the baseline questionnaire. The study will be carried out entirely on the internet and will not be blinded due to the nature of the intervention.

Junior physicians will be randomly allocated to one of two groups, namely, (1) the intervention group or (2) the control group. The intervention group will be provided with a link to download the Shift mHealth app; the participants in this group will be asked to use the app for a period of 30 days. The control group will be placed on a waitlist and will be asked to use the app after 3 months. Participants allocated to the control group are provided with a factsheet that lists several mental health support services, which they are encouraged to contact for further support. These participants will gain access to Shift after 3 months and will be asked to use the app for 30 days.

Primary and secondary outcomes will be measured through self-assessment using questionnaires at 3 time points: the first distributed as part of the baseline assessment, the second distributed 30 days after the baseline assessment, and the third distributed 3 months after the baseline assessment. Waitlist control participants will be further asked to respond to a questionnaire measuring primary and secondary outcomes after 4 months following the baseline assessment or 1 month after they gain access to the app. This will enable us to compare the 30-day app use patterns of the control group with those of the intervention group who used the app in the first month of the trial.

### Study Setting and Recruitment

Data collection will occur between January and August 2024; recruitment will occur using 3 strategies. Firstly, participants will be recruited through the Black Dog Institute’s web-based and social media channels including webpages on the Black Dog Institute website and targeted social media advertisements on Facebook (Meta Platforms, Inc) and Instagram (Meta Platforms, Inc). Secondly, the NSW Ministry of Health will disseminate study information through NSW Health public health systems. NSW is the state in which the greatest proportion of junior physicians in Australia are employed, primarily through public hospitals. Thirdly, the research team will partner with private medical colleges and other medical specialist organizations based in Australia to disseminate study information on medical platforms. Promotional materials may include the logos of the Black Dog Institute, the University of NSW Sydney, and NSW Health.

The inclusion criteria for participants taking part in this study include the following: (1) The participant must be currently working as a junior physician (ie, intern, hospital medical officer, resident medical officer, nonaccredited trainee, postgraduate trainee, principal house officer, registrar, or specialist trainee) in Australia. (2) The participant must own an internet-enabled smartphone that has an iOS (Apple) or Android (Google) operating system. (3) The participant must provide consent to participate.

Candidates who do not meet all the 3 eligibility criteria will be excluded from participation. Instead, they will be provided with mental health support options and information on other current research projects run by the Black Dog Institute.

The study is conducted remotely and occurs entirely on the web for all participants. This means that there is no direct contact with the research team unless a participant voluntarily contacts the team using contact details in the participant information statement and consent form.

### Intervention

**Figure 1:**
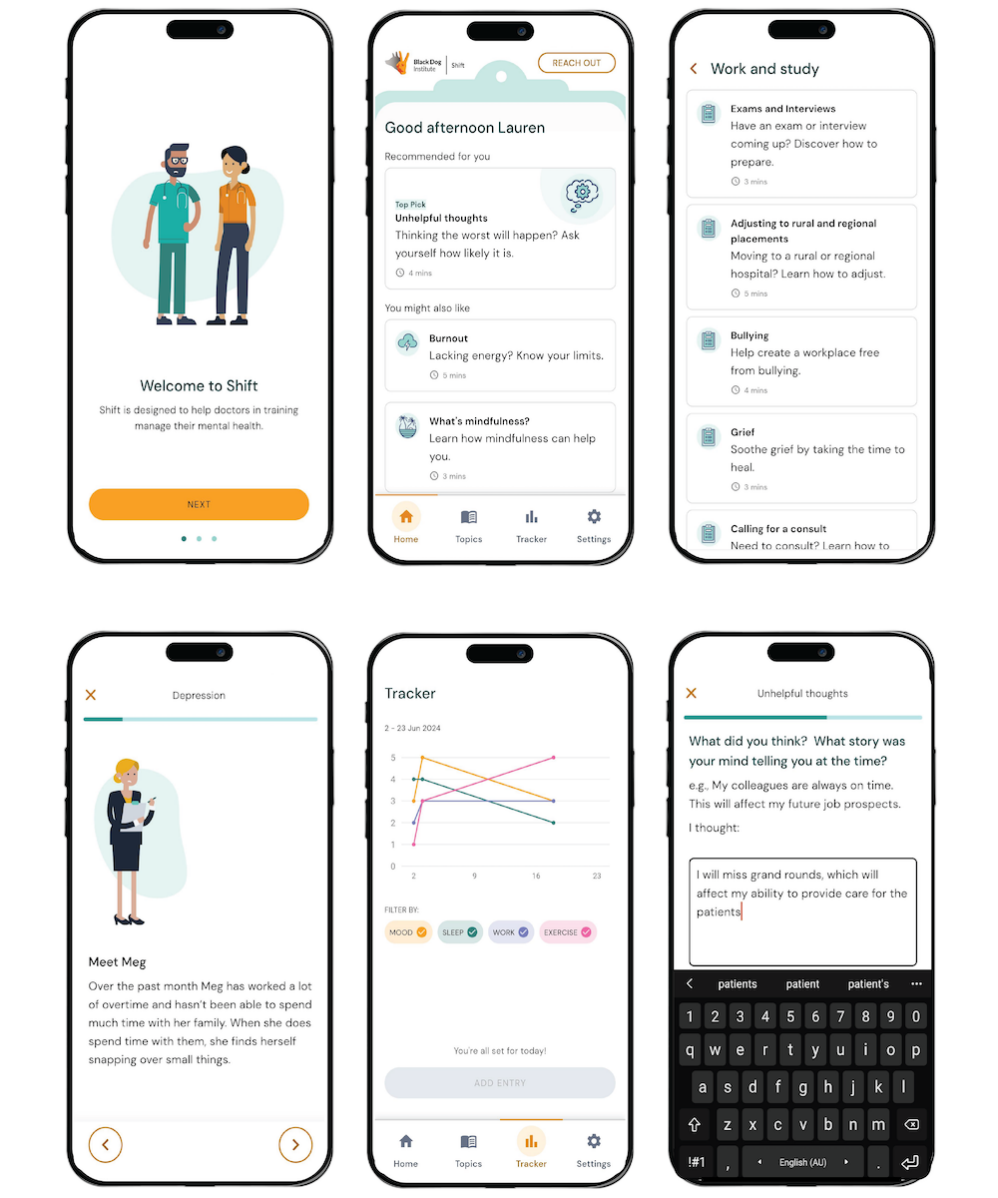
Visual examples of content in the Shift mobile health smartphone app for junior physicians.

The Shift modules include content covering topics related to mental health, getting support, lifestyle, and work. Other components of the Shift app include a tracking function for monitoring mood, physical activity, work-life balance, and sleep. The contents of the app are delivered through video, audio, graphical representation, and text displays. The module content was developed by the Black Dog Institute and was informed by recognized, evidence-based psychological approaches including cognitive behavioral therapy, acceptance and commitment therapy, mindfulness, and stress management. Thus, the app provides therapeutic, psychoeducational content and strategies contextualized for the specific needs of junior physicians. Examples of the most recent Shift app design and contents can be seen in Figure 1.

During the onboarding phase, the participants are encouraged to complete 2 to 5 activities per week, with each activity requiring 2 to 10 minutes from start to finish. However, participating junior physicians are free to decide how much they engage with the app and which modules they complete. Modules do not need to be completed in a fixed order, and a user can complete one module multiple times. At the onboarding phase, the participants are presented with the option to enable daily push notifications to use the app. They also have the option to change the time of the notifications or turn the notifications off at any time in the “settings” section of the app.

Several updates have been made to the Shift app since the uncontrolled trial of the app in 2020 [14]. This means that the Shift app available for the current RCT is version 2.0. Three new modules were added addressing financial difficulties, academic pressure, and common stigmatizing beliefs about physicians with mental health concerns. The app content was also modified to suit physicians working in all Australian states and in a postpandemic working environment. Changes were also made to enhance the user experience based on previous user feedback. For example, aesthetic updates were made to streamline the content, and in-app personalization was increased. A quality assurance phase by the development team ensured that these changes did not impact the quality or functionality of the app.

### Primary and Secondary Outcomes

#### Depression

The primary outcome of the study, *depression*, will be measured using the Patient Health Questionnaire-9 (PHQ-9), a reliable and valid 9-item screening tool for assessing depression severity during the past fortnight [15]. On a scale ranging from 0 (not at all) to 3 (nearly every day), respondents indicate their agreement with items describing common symptoms observed in major depressive disorder. Sum scores range from 0 to 27, with scores of 10 or higher indicative of a possible major depressive disorder. The PHQ-9 also includes a single item assessing the level of difficulty in daily functioning due to depressive symptoms. The validity and reliability of the PHQ-9 have been verified, and the measure has been used in physician populations [15,16].

#### Anxiety

The secondary outcome *anxiety* will be measured using the Generalized Anxiety Disorder-7 (GAD-7) Scale. The GAD-7 is a reliable and valid 7-item screener for generalized anxiety symptoms [17]. On a scale ranging from 0 (not at all) to 3 (nearly every day), respondents indicate their agreement with items describing common symptoms observed in general anxiety disorder. Overall scores range from 0 to 21, with scores of 10 or higher indicative of a possible general anxiety disorder. GAD-7 also includes a single item assessing the level of difficulty in daily functioning due to anxiety-related symptoms. The validity and reliability of GAD-7 have been confirmed, and the measure has been used in physician populations [17-19].

#### Work and Social Functioning

The secondary outcome *work and social functioning* will be measured using the 6-item Work and Social Adjustment Scale. The Work and Social Adjustment Scale is a valid and reliable tool used to measure functional impairment in private and professional areas of life [20]. On a scale ranging from 0 (not at all) to 8 (very severely), respondents indicate to what degree their ability to perform in social and work domains is impaired due to their mental health problems. Higher scores indicate a greater level of impairment in respondents’ ability to perform in their usual capacity.

#### Help Seeking

The secondary outcome *help seeking* will be measured using a brief 2-item assessment modeled after the General Help Seeking Questionnaire. The measure will assess the participants’ willingness to seek help for mental health concerns and will assess whether they recently sought help for mental health-related issues [21]. The respondents indicate their willingness to seek help on a Likert-type response scale ranging from 1 (*extremely unlikely*) to 7 (*extremely likely*) and indicate whether they sought help from a mental health professional in the previous month (using a simple *yes* or *no* response scale).

#### Quality of Life

The secondary outcome *quality of life* will be measured using the 5-item Linear Analog Scale Assessment. The Linear Analog Scale Assessment is a reliable and valid tool to measure the quality of life with respect to a number of domains (physical, emotional, spiritual, intellectual, and overall) [22]. On a scale ranging from 1 (as bad as it can be) to 10 (as good as it can be), the respondents are asked to rate their wellbeing during the past week.

#### Burnout

The secondary outcome *burnout* will be measured using the 16-item Oldenburg Burnout Inventory. The Oldenburg Burnout Inventory is a valid and reliable tool to measure the 2 main dimensions of burnout: exhaustion and disengagement from work [23]. Respondents use a 4-point Likert-type scale to indicate their degree of agreement with a number of questions relating to their level of burnout at work. Higher scores indicate a higher level of burnout.

#### App Acceptability

After using the app for 30 days, the participants will be asked to respond to 12 open- and closed-ended questions to rate the degree of satisfaction with the app components and identify areas of possible improvement in functionality and design. Seven items use a 5-point Likert-type scale. Using these items, the participant are asked to indicate how easy the app was to use, how helpful various components of the app were, whether they would use the app in the future, and whether they would recommend the app to other physicians in training. Furthermore, 4 open-ended questions assess which activities the participants found most and least helpful and whether the participants have general suggestions or the additional features they would like in the app. One final closed-ended question clarifies why the participants stopped using the app, if this was the case. Additionally, 1 optional item will seek clarification on the reasons why the participants chose to take part in the Shift study.

#### App Use

The app will automatically record use data such as the number of logins, time spent on app contents, and how many times the tracker function was used (to track mood, exercise, sleep, and work-life balance).

A timeline of all points of measurement for these outcomes is outlined in Table 1.

**Table 1:** Constructs and information assessed using web-based surveys and app data collection.

Outcomes	Time point 1: baseline survey	Time point 2: follow-up survey 1	Time point 3: follow-up survey 2	Time point 4: follow-up survey 3^c^	In-app data collection
Demographics	✓^a^	N/A^b^	N/A	N/A	N/A
Depression (PHQ-9^c^)	✓	✓	✓	—^d^	N/A
General anxiety disorder (GAD-7^e^)	✓	✓	✓	—	N/A
Work and social functioning (WSAS^f^)	✓	✓	✓	—	N/A
Help seeking (self-generated questions)	✓	✓	✓	—	N/A
Quality of life (LASA^g^)	✓	✓	✓	—	N/A
Burnout (OLBI^h^)	✓	✓	✓	—	N/A
App use and study-related questions (self-generated questions)	N/A	X^i^	N/A	—	N/A
App use data	N/A	N/A	N/A	N/A	✓

^a^For intervention and control group.

^b^N/A: not applicable

^c^PHQ-9: Patient Health Questionnaire-9.

^d^For control group only.

^e^GAD-7: Generalized Anxiety Disorder-7 Scale.

^f^WSAS: Work and Social Adjustment Scale.

^g^LASA: Life Linear Analog Scale Assessment.

^h^OLBI: Oldenburg Burnout Inventory.

^i^For intervention group only.

### Procedure

**Figure 2:**
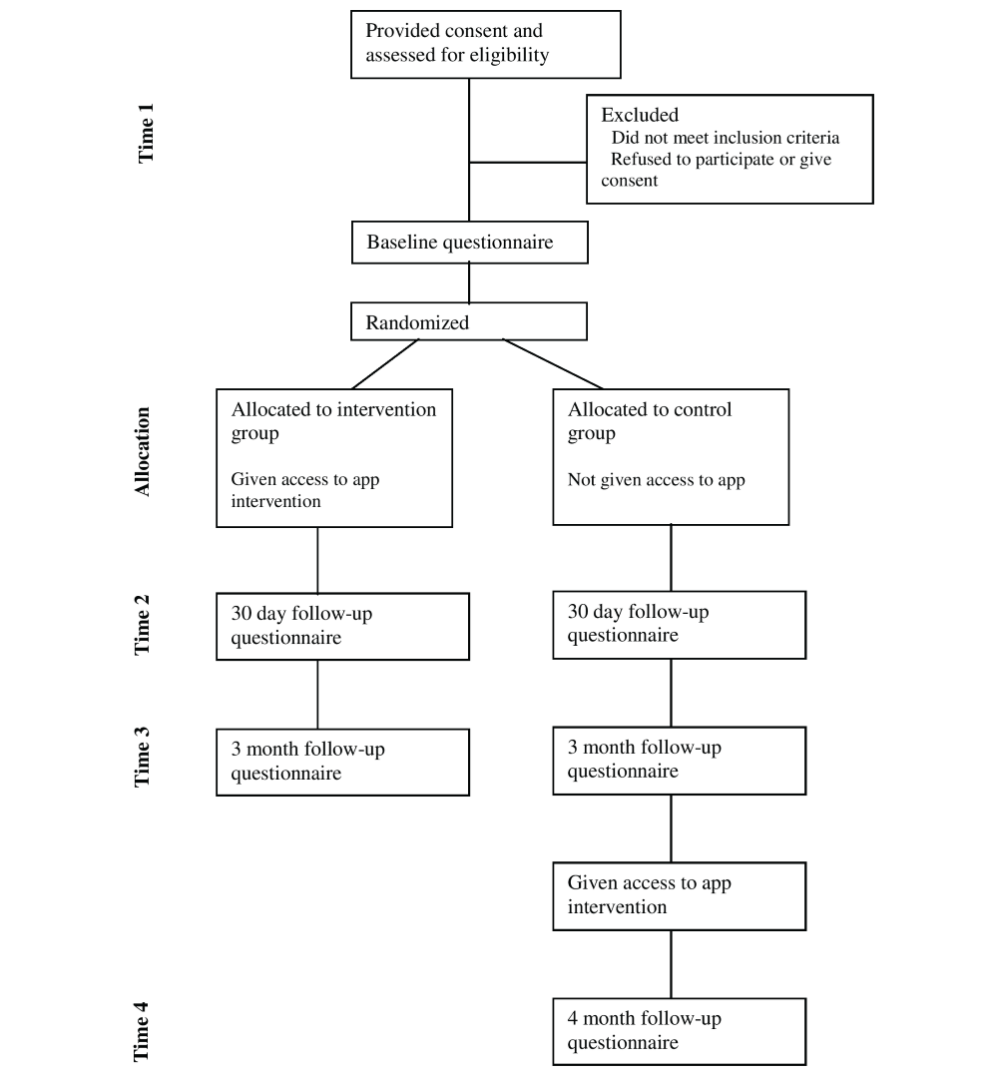
Flowchart describing how individual participants will progress through the phases of the research.

The procedure for the Shift RCT is depicted in Figure 2. At the study sign-up, candidates will be invited to visit a study-specific website [24]. Through the website, they will be presented a brief overview of the study purpose and the full participant information statement and consent form. The candidates who are willing to participate will be able to self-screen for eligibility. All the eligible participants will then be presented with a registration page in which they will be asked to input their first name, email address, mobile number, and password.

Following registration, the participants will be asked to complete a survey assessing the primary outcome (depression) and secondary outcomes (anxiety, work and social functioning, quality of life, burnout, and help-seeking intentions and behaviors). After completing the baseline survey, the participants will be randomly assigned to (1) the intervention group (in which a link will be provided through which they can download and use the app, immediately after registration) or (2) the waitlist control group (the members of will be added to a waitlist to use the app). The participants in the waitlist control group are provided with a factsheet listing contact details for several mental health support services and are encouraged to reach out if they would like further support. After the 3-month study period, the participants in the control group will also gain access to the app and will be asked to use it for 30 days. After the participants are provided with a link to download the Shift app on their smartphone, they will be able to use the app free-of-cost.

The primary and secondary outcomes will be measured in both the control group and the intervention group at a number of time points: at baseline and registration (time point 1), 30 days after registration (time point 2), and 3 months after registration (time point 3). These outcomes will also be measured among participants in the control group after 4 months following registration (time point 4), that is, once the participant has had access to the app for 30 days. At each follow-up measurement time point, the participants will receive an initial email reminder to complete the study. This will be followed by an email, a text reminder after 2 days and a text reminder after 9 days.

The study will run for a total of approximately 8 months, between January and August 2024. For participants in the intervention condition, the duration of the study period is 12 to 14 weeks. The duration of the study period for waitlist control participants is 16 to 18 weeks.

After the study is completed, participants can continue to use the app for as long as they wish. The participants can remove the app at any time by uninstalling it from their smartphone. In future, the app may be removed from the app store for development purposes; during this time, all users will be notified and will lose access to the app.

### Data Management

#### Data Management and Monitoring

All study data will be collected and recorded electronically through the Black Dog Institute Research Engine. The servers are hosted in a secure environment (Amazon Web Services) managed by the University of NSW Sydney.

The raw data contain identifiable information (ie, first name, private email address, and mobile phone number) from the registration section at baseline as well as an individual identifier code, which is automatically generated by the system for each participant. Direct individual identifiers will be removed during the transcription process before data preparation and analysis.

Every week, the research team will manually back up all data during the data collection phase; the team will also check that the data are properly archived during every quarter. The backups will be stored in a secure and password-protected University of NSW OneDrive (Microsoft Corporation) folder.

Only the research team approved by our ethics boards (University of NSW Human Research Ethics Committee and Sydney Local Health District Ethics Review Committee) and the designated Black Dog Institute technical staff can access the data. The data will be retained for a minimum of 15 years after the publication of the research findings.

#### Trial Harms and Risk Management

Because the study addresses common mental health concerns and challenges associated with being a junior physician, there is a possibility that a few participants may find it distressing to engage with this content. We do not, however, expect the app itself to result in more serious harm, and there were no adverse or serious adverse events registered in the preceding app trials [14]. We define adverse events as any event in which a participant contacts the Shift research team with the indication that the app has had a harmful effect on their health, including any level of psychological distress. The principal investigator will keep a record of all adverse events and inform both the approving ethics committees (the University of NSW Human Research Ethics Committee and the Sydney Local Health District Ethics Review Committee) within 24 business hours following an adverse event. A data safety monitoring board will review the safety protocol and risk management plan. If any risks or adverse events emerge during the trial, the board will meet to review the continuing safety of the study. The board will consist of 3 reviewers who are not associated with the RCT and have a clinical background. If a serious adverse event or reaction is reported, or several adverse events or reactions, the study’s data safety monitoring board will review the impact of the study on the participants, and the study may be terminated if the board determines the study is harmful to participants.

When participants initially access the study website, they are presented with the participant information statement and consent form in which the study risks are outlined and emergency helpline contact details are provided. In this form, participants are also provided with the contact details of the research team. If participants contact the research team expressing any level of psychological distress or requesting further support, the team will arrange a callback from the Black Dog Institute’s Clinical Services to ensure that appropriate care is being offered to the participant.

On every page of the study website, including the web-based questionnaires, a banner with emergency-contact details is presented at the bottom of the page. These details can be used by the participants if they are experiencing psychological distress. Furthermore, the participants who have medium or high scores (suggesting medium or severe ranges for depression and anxiety) or indicate having suicidal thoughts while responding to the depression measure will see a pop-up window that informs them of their scoring result along with suggestions on how to seek help through a designated helpline. They are also advised to consider making an appointment with their general practitioner or a mental health professional if their symptoms increase or persist.

The app features an in-built “reach out” button at the top right-hand corner of the home screen to support participants who may become distressed while using the app. This button takes users directly to contact details for mental health services and 24×7 telephone helpline numbers. Furthermore, the participants who score poorly on the daily mood checkups (those who select the response options “worse than usual” or “slightly worse than usual”) are provided with suggestions to access mental health support options.

### Data Analysis

#### Estimands and Statistical Analysis

##### Estimands

The primary estimand [25] will reflect intention-to-treat principles and will be based on all participants in the group to which they were randomized regardless of actual receipt, uptake of the intervention, alternative support or interventions received, and withdrawal from the study. A mixed-model, repeated measures analysis will be used for the primary outcome and continuously scaled secondary outcome variables to estimate and compare the change in mean PHQ-9 score in each intervention arm from time point 1 to time points 2, 3, and 4 (if applicable). This form of analysis assumes that unobserved outcome data are missing at random.

As substantial attrition is expected given the distal, universal nature of the intervention, a sensitivity analysis of the primary outcome will be undertaken if the outcome of the intention-to-treat is statistically significant. The “jump-to-reference” approach will be used [26]. This approach assumes that the participants who withdraw or have no postbaseline data have the same trajectories as participants in the control condition. This will yield a robust, conservative lower bound estimate of the intention-to-treat effect in the presence of missing outcome data.

Secondary estimands will estimate the treatment effect in participants who have engaged with the Shift app to various extents. Given that app users are free to choose the modules they use and to access them as often as desired, the concept of full compliance is not applicable. One estimate will involve participants who complete the recommended 8 or more modules during the intervention. The second estimate will broaden engagement to participants who have completed at least 4 modules. This reflects the levels of engagement more commonly observed in the previous version of the app [14]. The complier average causal effect comparing *compliers* in the intervention group to *would-be compliers* in the control group will be estimated using latent variable methods [27]. A conventional per-protocol analysis, including only participants allocated to the intervention group who have downloaded the app, will also be conducted.

##### Primary Outcome

The change in mean depressive symptoms will be assessed by a planned comparison of the difference between groups in the change of PHQ-9 scores from baseline to postintervention using a linear mixed model for repeated measures. This test will be undertaken with an α of .05. Models will include factors of intervention arm (intervention or control group), occasion of measurement (time points 1, 2, and 3), and their interaction. An unstructured residual variance-covariance matrix will accommodate within-participant dependency. Tests of significance will use the Kenward-Roger method of df adjustment based on the observed information matrix. Where necessary, transformation of the outcome variable will be undertaken to ensure the distributional assumptions of the model are met.

Depression caseness will be estimated using the established threshold for moderate depressive symptoms using the PHQ-9 total score (a score of 10 or greater). Odds ratios and predicted probabilities will be obtained from a generalized logistic mixed model, which will include random participant intercepts and the factors used in linear models. This will allow us to determine the app’s effectiveness in reducing symptomatic status in participants who are likely to have depression, as well as investigate whether the participants who are mentally healthy were able to stay well after the app use.

##### Secondary Outcomes

Group differences on secondary outcomes on the GAD-7, Work and Social Adjustment Scale, Linear Analog Scale Assessment, and Oldenburg Burnout Inventory scales will follow the same analysis strategy that is used for the primary outcome. For the categorical secondary outcome measure (modified General Help Seeking Questionnaire), analogous generalized mixed models will be used.

Secondary analyses will also include changes in the primary and other outcome variables from baseline (time point 1) to follow-up (time point 3) to establish retention of any benefit. The waitlist group will be provided with access to the intervention after follow-up at time point 3. Postintervention outcomes in the waitlist group (time point 4) will be compared with the intervention group’s postintervention (time point 2) scores to estimate changes after they have accessed the intervention. Subject to qualifications arising due to attrition and any natural drift with the passage of time, this analysis will stand as a quasi-replication of the primary outcome of the trial.

As participant sample size allows, a priori defined subgroup analyses (focusing on age, sex, specialty, training level, and location) will be conducted to explore how the primary and secondary outcomes are affected by app use, baseline symptoms patterns (severity of depression and anxiety symptoms), and related factors (eg, self-reported wellbeing status) across all examined time points to inspect group and subgroup trajectories.

Qualitative, open-ended feedback responses will be examined to aid subsequent app changes and future study design considerations. Individual data collected in the questionnaires will be assessed using a structured tabular thematic analysis approach for brief texts, as outlined in Robinson [28].

##### Sample Size

We determined a total sample size of 778 research participants. Power calculations were based on a recent systematic review and meta-analysis quantifying the efficacy of digital prevention interventions for depression [29]. Hence, an estimated Cohen *d* effect size of 0.25, an α value of .05, a power of 0.8, and a correlation coefficient of 0.6 yielded the required sample size of 404, without any participant dropouts.

A systematic review and meta-analysis examining dropout rates during clinical trials of smartphone apps for depressive symptoms found a pooled dropout rate of 48% [30]. Therefore, we expect to randomize a total of 777 participants (N=404+373) to the 2 trial conditions to conduct a sufficiently powered analysis. To allow for evenly sized groups, this number was rounded off to 778 participants.

##### Data Exclusion

The participants who withdraw and do not wish for their data to be used will be removed from the analysis.

### Ethical Considerations

The trial is registered in the Australian and New Zealand Clinical Trials Register (ACTRN12623000664640) and was approved by the University of NSW Human Research Ethics Committee (HC230355) and the Sydney Local Health District Ethics Review Committee (RPAH Zone, X23-0424). Two ethical approvals were obtained to enable the research team to recruit through nongovernment and government organizations. This is because recruitment through hospitals in Australia requires ethics approval with a government-run human research ethics council such as the Sydney Local Health District Ethics Review Committee.

All candidates will be required to provide informed consent to participate in the study. This is done by digitally signing a web-based participant information statement and consent form before commencing participation in the trial. Candidates are free to withdraw their consent at any time during or after their participation in the trial. Furthermore, all data used in the study analysis will be deidentified.

After the completion of each of the follow-up questionnaires, the participating junior physicians will have the option of participating in a draw to win 1 of 7 eGift vouchers worth Aus $100 (US $68.06) or Aus $200 (US $136.11).

## Results

### Overview

The study was funded from November 2022 to December 2024 by the NSW Ministry of Health. Data collection ran from January to August 2024, during which time 780 participants enrolled in the study. As of September 2024, data analysis was underway, with results expected to be submitted for publication in 2025.

### Dissemination

Results will be disseminated through open-access, peer-reviewed journal articles, professional and media publications, and presentations at scientific conferences. Results may be shared with specialist bodies and forums related to junior physicians and may be included in training materials for physicians. A summary of the results and a copy of any publications from the study will also be made available to participants on request. Participating individuals will not be identified when disseminating the study findings.

## Discussion

### Summary

Workplace stressors contribute to poor mental health outcomes in junior physicians. These risk factors include long working hours, high work demands, unsupportive work environments, and reluctance to seek help due to concerns regarding confidentiality and potential professional repercussions [1-4]. In light of this, the Shift smartphone app was created to support the mental health of junior physicians in a manner that is entirely confidential, easily accessible, and available 24×7. This paper describes the protocol for an RCT to test the effectiveness of the Shift app in reducing symptoms of depression and other mental health outcomes among junior physicians who used the app. While previous studies have found that mHealth apps can reduce depression and provide evidence-based mental health support for other workforces who are at risk [9,10,31], to the best of our knowledge, the effectiveness of a mHealth app for junior physicians has not yet been evaluated in a randomized controlled design.

### Strengths

The Shift app specifically targets common problems and concerns faced by junior physicians. During the design phase of the app, qualitative interviews with 12 junior physicians showed that there was a lack of information and support for the unique issues faced at the early stages of a physician’s medical career, including navigating professional hierarchies, occupational stress, and the high amount of emotional labor [32]. Therefore, the Shift app’s content was designed to be specific to junior physicians, with modules incorporating stories of various junior physicians facing work-related or emotional problems. This was validated by a rigorous codesign process to ensure that the design, wording, and the information presented were suitable and relatable for junior physicians who use the app. Furthermore, the app was designed to address the common barriers to engaging with mental health support tools, as identified by junior physicians [13]. Because junior physicians are time restricted and required to work irregular hours, these barriers include confidentiality, ease of use and ease of access.

Furthermore, as the participants were asked to use the app freely within the 30-day intervention period, we do not expect significant variations between app use patterns in the RCT compared with app use patterns outside of an RCT setting.

### Limitations

One limitation of this study is that the study participants will not be blinded with regard to their respective group allocation (intervention or control); nor will they be blinded to the suspected outcomes. This is for feasibility and transparency reasons; however, the potential for bias must be recognized. It is possible that knowledge of group allocation and outcomes is subjected to demand characteristics. While this is an important limitation of the research design, this approach aligns with the self-guided nature of the Shift app in real-world settings.

It is possible that the app will be mostly beneficial to a subset of participants with a low-to-medium level of mental health knowledge, due to the universal nature of the intervention and content. External validity concerns may be further exacerbated by potentially high rates of study attrition. This is because junior physicians typically have limited time to engage with nonessential tasks and, thus, may not return to complete all follow-up assessments or engage with the app on a regular basis or with enough frequency or duration to obtain the intended benefit. Furthermore, because participants will use the app intervention without further direction after the initial in-app onboarding, app use will not be standardized. It was found in the pilot testing phase of the Shift app that our target audience (junior physicians) disliked the highly structured design of the app and preferred to complete the modules that interested them personally [13]. Therefore, while this open app design is suited to physicians’ preferences, we expect considerable variation in the app contents that participating junior physicians engage with and how frequently the app is used during the trial phase.

It must also be noted that although the trial does not screen candidates on the basis of their mental health symptom severity, it is possible that not all levels of symptom severity will be represented in the final sample. For example, it is possible that physicians who are already seeking support for mental health problems are more inclined to sign up for this research. Consequently, any inferences drawn will be limited to the mental health spectrum represented in the study sample and may not be generalizable to junior physicians based in Australia as a whole.

Finally, many challenges faced by junior physicians are related to poor workplace factors, such as long work hours and hierarchical work environments. Participants’ individual organizational cultures, existing mentoring structures, and sets of occupational risk factors will constitute important institutional influences on physicians’ mental health and wellbeing. Qualitative interviews with junior physicians revealed that the main factors impacting their mental health were (1) professional hierarchies, (2) occupational stress, (3) emotional labor, and (4) continuation of worry and rumination at home [32]. While the current intervention is targeted at addressing the influence of workplace stressors on mental health experiences and reducing worry and rumination, the app is largely focused on individual-level strategies and on how to seek mental health support through professional and private avenues. Systematic, organizational-level workplace change is likely required in conjunction with self-guided digital interventions to create a lasting improvement in mental health outcomes for junior physicians.

### Conclusions

The Shift app is the first intervention of its kind created to support the mental health of junior physicians. If the app is found to be effective and suitable for junior physicians, it will pave the way to providing a widely accessible tool to increase help-seeking behaviors and serve as a preventative tool in the high-stress work environment in which junior physicians operate. However, the intervention must undergo rigorous trials, given that many digital interventions currently available for mental health are recommended without testing and may be ineffective [33].

In the current postpandemic era, the medical workforce has been placed under increasingly heightened levels of stress. An efficacious mHealth app tool would act as a first step toward increasing awareness about mental health and promoting avenues through which users can seek help, fostering a more open and informed environment for junior physicians.

## Abbreviations

GAD-7: Generalized Anxiety Disorder-7 Scale
mHealth app: mobile health smartphone app
NSW: New South Wales
PHQ-9: Patient Health Questionnaire-9
RCT: randomized controlled trial

## Data Availability

The data sets generated during and analyzed during this study are available from the corresponding author on reasonable request.
